# Vitamin C Inhibits Blood-Stage *Plasmodium* Parasites via Oxidative Stress

**DOI:** 10.3389/fcell.2021.639944

**Published:** 2021-05-11

**Authors:** Xiaoyu Shi, Meng Wei, Zihao Xu, Ying Liu, Mujia Zhang, Li Lv, Qian Wang

**Affiliations:** ^1^Department of Immunology, School of Basic Medical Sciences, Key Laboratory of Immune Microenvironment and Diseases of Educational Ministry of China, Tianjin Key Laboratory of Cellular and Molecular Immunology, Tianjin Medical University, Tianjin, China; ^2^Department of Endocrinology and Metabolism, Tianjin Medical University General Hospital, Tianjin, China; ^3^National Laboratory of Biomacromolecules, Institute of Biophysics, Chinese Academy of Sciences, Beijing, China

**Keywords:** vitamin C (ascorbic acid), *Plasmodium*, glucose transporter, oxidative stress, eryptosis

## Abstract

During the *Plasmodium* erythrocytic cycle, glucose is taken up by glucose transporters (GLUTs) in red blood cells (RBCs) and supplied to parasites via the *Plasmodium* hexose transporter. Here, we demonstrate that the glucose uptake pathway in infected RBCs (iRBCs) can be hijacked by vitamin C (Vc). GLUTs preferentially transport the oxidized form of Vc, which is subsequently reduced in the cytosol. Vc, which is expected to burden the intracellular reducing capacity, inhibits *Plasmodium berghei* and *Plasmodium falciparum* growth. Vc uptake is drastically increased in iRBCs, with a large proportion entering parasites. Increased absorption of Vc causes accumulation of reactive oxygen species, reduced ATP production, and elevated eryptosis in iRBCs and apoptosis in parasites. The level of oxidative stress induced by Vc is significantly higher in iRBCs than uninfected RBCs, not seen in chloroquine or artemisinin-treated iRBCs, and effective in inhibiting chloroquine or artemisinin-resistant parasites. These findings provide important insights into the drug sensitivity of *Plasmodium*.

## Introduction

Usage of vitamin C (Vc) has been suggested in cancer therapy (Cameron and Pauling, 1976, 1978; Chen et al., 2007, 2008). Humans and a few high vertebrates are not capable of synthesizing Vc (Nakajima et al., 1969; Pollock and Mullin, 1987); it is absorbed via sodium Vc cotransporters (SVCTs) (Vera et al., 1993; Tsukaguchi et al., 1999) and glucose transporters (GLUTs), namely, the GLUT family (Wood and Trayhurn, 2003), due to the chemical similarity between Vc and glucose. GLUTs prefer the oxidized form of Vc, dehydroascorbic acid (DHA), for transportation (Yun et al., 2015). Inside the cytosol, DHA is quickly reduced by nicotinamide adenine dinucleotide phosphate (NADPH) (Linster and Van Schaftingen, 2007). Recent studies have revealed that the selective impact of high-dose Vc on colorectal cancer cells is attributed to the high demand for glucose during the tricarboxylic acid (TCA) cycle and glycolysis transition in these cells (Yun et al., 2015). Glucose transporter 1 (Glut1) is often up-regulated in growing tumor cells (Altenberg and Greulich, 2004). The elevated glucose uptake can be taken over by Vc when present in large amounts. Subsequently, the increase in transported DHA decreases the intracellular reducing power and causes oxidative stress that kills the tumor cells (Yun et al., 2015).

We hypothesize that the same strategy can be applied to selectively inhibit the growth of *Plasmodium-*infected red blood cells (RBCs). Because of a lack of nuclei and organelles, mature RBCs rely entirely on glucose for energy (van Wijk and van Solinge, 2005). Glut1 is highly expressed on the surface of human RBCs and Glut4 on rodent RBCs (Montel-Hagen et al., 2008a, b). *Plasmodium* parasites, the causative agent of malaria, also require a continuous supply of glucose for survival (Foth et al., 2005; Mancio-Silva et al., 2017). Glut1 activity was recently shown to be critical for liver infection by the parasites (Meireles et al., 2017). *Plasmodium* hexose transporter (HT), which absorbs glucose for the parasites, is also essential (Woodrow et al., 2000) and has been proposed as an antimalarial drug target (Joet et al., 2003). The demands for glucose from both RBCs and the parasites are expected to be even higher during blood-stage development of *Plasmodium*. In addition, GLUTs may be the dominant pathway by which Vc enters erythrocytes, as these cells lose their SVCTs during maturation (May et al., 2007). Although most nutrients can also be transported into infected RBCs (iRBCs) through the plasmodial surface anion channel (PSAC) on the infected cell membrane (Desai et al., 2000), sugars are transported into iRBCs predominantly through human RBC’s endogenous carriers (Kirk et al., 1996). Vc likely takes advantage of the glucose permeation pathway and has an increased impact on *Plasmodium*-iRBCs than cancer cells or other normal cells.

## Results

### Vc Inhibits Blood-Stage Growth of *Plasmodium berghei in vivo*

**FIGURE 1:**
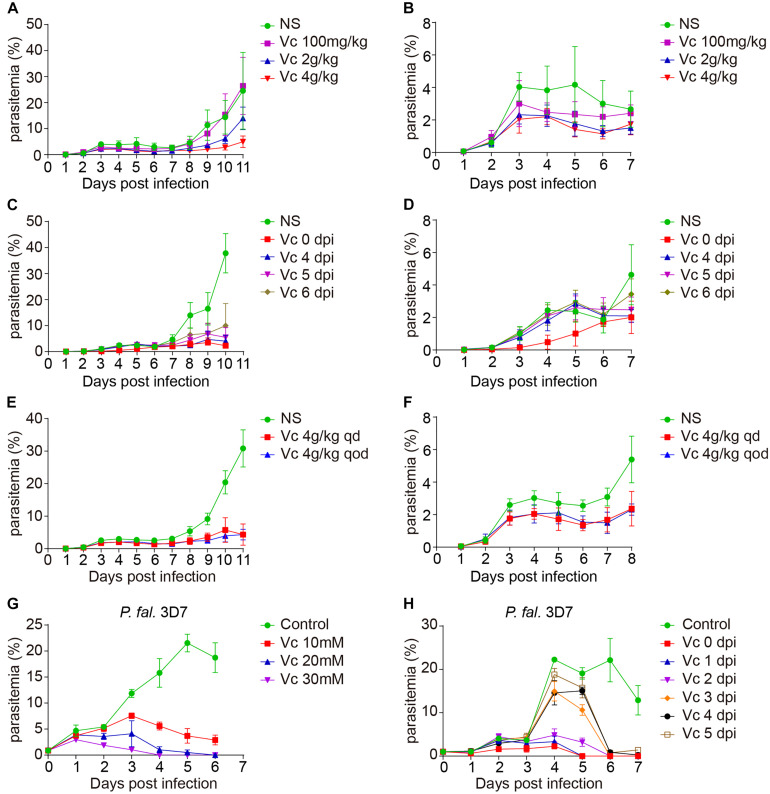
Vitamin C inhibits *Plasmodium* growth. **(A)** Vc (4 g/kg) inhibited *P. berghei* ANKA growth in BALB/c mice (*n* = 10–11 mice/group). Normal saline (NS) or Vc was injected intraperitoneally once a day from day 0. NS vs. 100 mg/kg Vc: n.s., NS vs. 2 g/kg Vc: *P* < 0.0001, NS vs. 4 g/kg Vc: *P* < 0.0001, 2 g/kg Vc vs. 4 g/kgVc: *P* < 0.01. **(B)** Parasitemia on days 1–7 p.i. in the mice from **(A)**. **(C)** Vc treatment inhibited parasite growth when administered from days 4 to 6 p.i. (*n* = 4–9 mice/group). Similar to **(A)**, 4 g/kg Vc was injected once a day starting from different time points (days 0, 4, 5, and 6 p.i.). NS vs. Vc treatment from days 0/4/5/6 p.i.: *P* < 0.0001. **(D)** Parasitemia on days 1–7 p.i. in the mice from **(C)**. **(E)** Vc treatment was also effective when the administration interval was extended (*n* = 8 mice/group). The frequency of Vc administration was once every day (qd) or once qod. NS vs. Vc 4 g/kg qd/qod: *P* < 0.0001; Vc 4 g/kg qd vs. Vc 4 g/kg qod: n.s. **(F)** Parasitemia on days 1–8 p.i. in the mice from **(E)**. **(G)** Vc inhibited blood-stage *P. falciparum* 3D7 growth *in vitro*. *P. falciparum* 3D7 was cultured *in vitro* with a starting parasitemia of 1% at day 0 (*n* = 3/group). The indicated dose of Vc was added to the media for 3 h daily from day 0 and parasitemia determined daily before Vc treatment. Control vs. Vc 10/20/30 mM: *P* < 0.0001. **(H)** 20 mM Vc inhibited *P. falciparum* 3D7 growth when administered from days 1/2/3/4/5 (*n* = 3/group). Control vs. Vc treatment from days 0/1/2/3/4/5: *P* < 0.0001. All data shown are mean ± SD and representative of three independent experiments. Two-way ANOVA with Tukey multiple comparisons was used to analyzed the data. n.s., not significant.

The dosage of Vc in malaria treatment has not exceeded 180 mg/kg of body weight in mice (Isah and Ibrahim, 2014). In contrast, the concentration of Vc for cancer cell killing usually reaches 20 mM in culture media or 4 g/kg of body weight by intraperitoneal (IP) injection. To verify whether Vc could inhibit the growth of blood-stage parasites, we treated infected mice daily with IP injection of various amounts of Vc. Mice with daily saline injection exhibited a typical parasite growth pattern over 10–11 days (initial peak of 3%–5% parasitemia followed by a rapid increase), whereas mice with daily Vc injection (4 g/kg) exhibited significantly reduced parasitemia during both the initial peaking and the latter proliferation (Figures 1A,B). A dose of 0.1 g/kg had no detectable effect, and a dose of 2 g/kg resulted in intermediate inhibition (Figures 1A,B). These results suggest that high doses of daily Vc efficiently prevent *Plasmodium berghei* infection.

To test whether Vc is still effective after infection is established, we started Vc treatment at different postinfection time points. Vc injections 4–6 days postinfection (dpi) efficiently prevented a rapid increase in parasitemia (Figures 1C,D). As expected, injections initiated 1–3 dpi were also effective in suppressing parasitemia (Supplementary Figures 1A,B). These data suggest that continuous Vc treatment not only prevents infection, but also inhibits existing infection. Next, we tested whether Vc would be effective when administered every other day (qod). Notably, similar parasitemia patterns were observed when high-dose Vc was injected into infected mice in an “every day” (qd) manner or qod (Figures 1E,F). Although Vc had a significant inhibitory effect on parasite growth, it cannot eliminate parasite infection alone (Supplementary Figure 1C). These results demonstrate that inhibition can be achieved with less frequent Vc injection.

### Vc Inhibits Blood-Stage Growth of *Plasmodium falciparum in vitro*

Next, we tested whether Vc also works on *Plasmodium falciparum* 3D7. When injected into mice at 4 g/kg, Vc reaches maximal serum concentrations of ∼40 mM within 3 h (Chen et al., 2008). Thus, we added Vc to *P. falciparum* strain 3D7 cultures for 3 h each day. Parasite growth was efficiently inhibited by the addition of 10, 20, or 30 mM Vc, with the 30 mM treatment being most effective (Figure 1G). When Vc was continuously added without replacing the existing media, maximal inhibition was achieved by 5 mM Vc (Supplementary Figure 1D). Finally, we added 20 mM Vc at various dpi. No obvious increase in parasitemia was observed with treatment 0–2 dpi, and treatment 5 dpi resulted in a decrease in parasitemia to barely detectable levels after 2 days (Figure 1H). Taken together, these results suggest that high-dose Vc effectively inhibits the growth of blood-stage *P. falciparum* 3D7.

### Vc Is Taken Up by Erythrocytes and Parasites Through GLUT and HT

**FIGURE 2:**
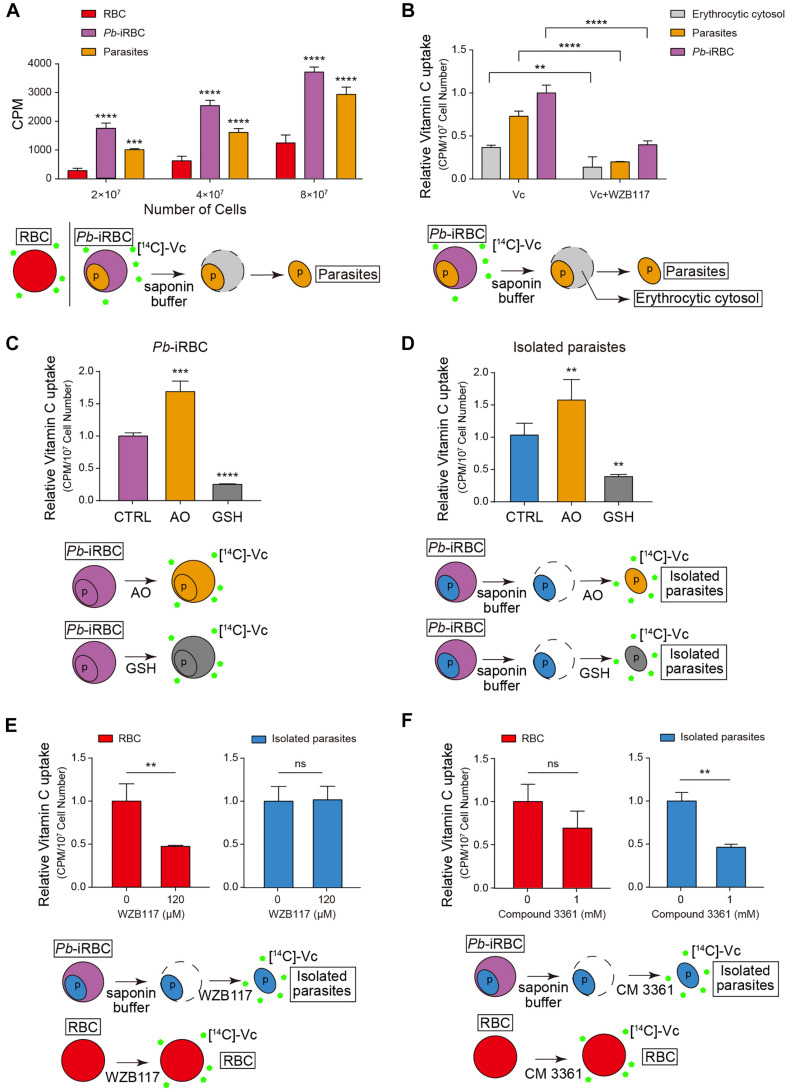
Vitamin C is taken up by erythrocytes and parasites through different transporters. **(A)** The amount of Vc taken up by iRBCs increased compared to uninfected RBCs, and Vc was also taken up by parasites in iRBCs. The uptake was measured in the presence of RPMI 1640 medium, which contains 2 g/L of glucose (∼11 mM). Data are mean ± SD and were analyzed by two-way ANOVA with Tukey multiple comparisons. **(B)** GLUT inhibitor WZB117 inhibited Vc uptake in iRBCs. Infected RBCs were pretreated with or without 120 μM WZB117 and detected the amount of Vc uptaken by iRBCs, isolated parasites, and erythrocytic cytosol, respectively. Uptake values (CPM) were normalized to cell number. Data are shown as mean ± SD and analyzed by two-way ANOVA with Tukey multiple comparisons. **(C)** Ascorbate oxidase (AO; 10 U/mL) increased Vc uptake in iRBCs, whereas GSH (10 mM) decreased Vc uptake. 2 × 10^7^ iRBCs were pretreated with PBS, 10 U/mL AO, or 10 mM GSH for 5 min and then incubated with [^14^C]-Vc to detect Vc uptaken. Relative [^14^C]-Vc uptake in the PBS-treated group was defined as 100%. Data are mean ± SD, and one-way ANOVA was performed. **(D)** AO increased Vc uptake in isolated parasites, whereas GSH decreased Vc uptake. Parasites isolated from 4 × 10^7^ iRBCs were pretreated with PBS, 10 U/mL AO, or 10 mM GSH, and the amount of Vc taken up was measured. Data are presented and analyzed as described in **(C)**. **(E)** WZB117 inhibited Vc uptake in normal RBCs but not in isolated parasites; 2 × 10^7^ RBCs or parasites isolated from 4 × 10^7^ iRBCs were pretreated with or without 120 μM WZB117 for 5 min before incubation with [^14^C]-Vc. Relative [^14^C]-Vc uptake in RBCs and parasites without WZB117 treatment was defined as 100%. Unpaired two-tailed *t* test was used. **(F)** *Plasmodium* hexose transporter (HT) inhibitor compound 3361 inhibited Vc uptake in isolated parasites but not in normal RBCs. 2 × 10^7^ RBCs or parasites isolated from 4 × 10^7^ iRBCs were pretreated with or without 1 mM compound 3361 for 5 min. Data are expressed and analyzed as described in **(E)**. All data are representative of at least three repetitions. **P < 0.01, ***P < 0.001, ****P < 0.0001, and n.s., not significant.

To monitor whether Vc is taken up by erythrocytes and *Plasmodium*, we used ^14^C-radiolabeled Vc ([^14^C]-Vc) to test its absorption in rodent erythrocytes and *P. berghei*. Isolated iRBCs took up much more [^14^C]-Vc than uninfected RBCs (Figure 2A). Interestingly, substantial amounts of [^14^C]-Vc were detected in parasites from iRBCs (Figure 2A), much more than in the cytosol of iRBCs (Figure 2B), suggesting a parasitic absorption mechanism. Vc has been suggested to pass through GLUT as DHA, an oxidized form of ascorbic acid. In contrast, nutrient diffusion into iRBC through the PSAC on host membranes (Nguitragool et al., 2011; Pillai et al., 2012) would not require modification. Consistent with a GLUT dependence, prior treatment with ascorbate oxidase (AO), which results in increased DHA, effectively boosted Vc absorbance by iRBCs (Figure 2C). Conversely, the addition of reducing agent glutathione (GSH) prevented absorbance (Figure 2C). These results suggest that Vc uptake by iRBCs is predominantly GLUT-dependent. Similar results were obtained when isolated parasites were tested (Figure 2D), suggesting selectivity of oxidized Vc for parasitic plasma membrane permeation.

The uptake of DHA was likely via rodent Glut4, as GLUT inhibitor WZB117 [2-fluoro-6-(m-hydroxybenzoyloxy) phenyl m-hydroxybenzoate] significantly reduced [^14^C]-Vc absorption by RBCs and iRBCs (Figures 2B,E). Glucose uptake by the parasites is mediated by a conserved *Plasmodium* HT (Woodrow et al., 2000), which can be inhibited by compound 3361 (Joet et al., 2003). Therefore, we tested whether Vc uptake by the parasites is through *P. berghei* HT. Isolated parasites took up [^14^C]-Vc efficiently (Figure 2B), which was partially blocked by compound 3361 [3-*O*-(Undec-10-en)-yl-D-glucose] (Figure 2F), but not at all by WZB117 (Figure 2E). In contrast, compound 3361 had a minor impact on [^14^C]-Vc uptake by RBCs (Figure 2F). [^14^C]-Vc was similarly taken up when *P. falciparum* 3D7–iRBCs were tested, but the total absorbance was blocked by both WZB117 and compound 3361 (Supplementary Figure 2). Collectively, these results suggest that intraerythrocytic parasites likely acquire Vc from the cytosol of iRBCs using *Plasmodium* HT.

### Vc Triggers Oxidative Stress in iRBCs and RBC-Hosted Parasites

**FIGURE 3:**
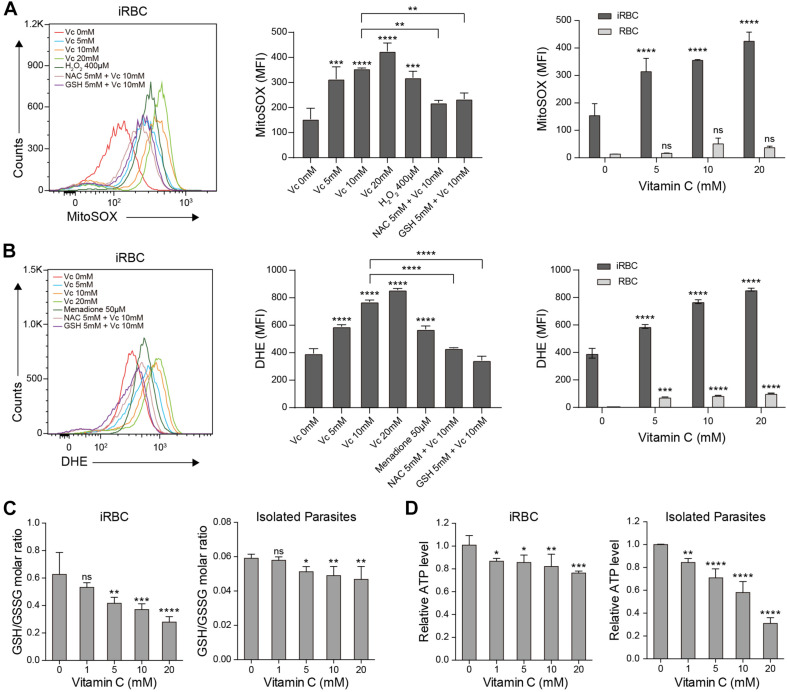
Vitamin C causes oxidative stress in *P. berghei–*infected RBCs and parasites. **(A)** Vc induced mitochondrial superoxide production in a dose-dependent manner in *P. berghei*–infected RBCs, which could be reversed by the antioxidants N-acetyl cysteine (NAC) and glutathione (GSH). Left: Representative FACS plots of superoxide production in iRBCs. Middle: Quantitative superoxide production in iRBCs (*n* = 3/group). One-way ANOVA was performed to detect statistical differences. Right: Quantitative superoxide production in both iRBCs and RBCs (*n* = 3/group). Differences between Vc-treated iRBCs/RBCs and non-treated iRBCs/RBCs were analyzed by two-way ANOVA with Tukey multiple comparisons. **(B)** Vc induced superoxide production in a dose-dependent manner in *P. berghei–*infected RBCs measured by DHE, which could be reversed by NAC and GSH. Left: Representative FACS plots of superoxide production in iRBCs. Middle: Quantitative superoxide production in iRBCs (*n* = 3/group). Right: Quantitative superoxide production in both iRBCs and RBCs (*n* = 3/group). **(C)** Vc treatment decreased the GSH/GSSG ratio in *P. berghei–*infected RBCs (left panel) and isolated *P. berghei* parasites (right panel) (*n* = 5/group). **(D)** Vc decreased ATP production in *P. berghei–*infected RBCs (left panel) and isolated parasites (right panel) (*n* = 5/group). Based on calibration, the ATP concentrations of the tested samples derived from iRBCs and isolated parasites treated with 0 mM Vc are 5.40 ± 0.33 μM and 0.72 ± 0.003 μM. One-way ANOVA was performed to analyzed the results in **(C, D)**. All data are presented as mean ± SD and are representative of at least three repetitions. *P < 0.05, **P < 0.01, ***P < 0.001, ****P < 0.0001, and n.s., not significant.

To test whether high-dose Vc causes the accumulation of reactive oxygen species (ROS), isolated iRBCs were incubated with Vc and measured by MitoSOX Red and dihydroethidium (DHE) staining. As expected, ROS gradually accumulated when the concentration of Vc increased (Figures 3A,B). To assess superoxide production by only the parasites, we measured Vc-treated iRBCs using MitoSOX Red, a mitochondria-targeting superoxide indicator that would not be optimized with RBCs because of a lack of mitochondria. Oxidation gradually increased with increased concentrations of Vc (Figure 3A). By contrast, Vc-treated RBCs had a detectable increase of MitoSOX or DHE signals, but it was not comparable to that in iRBCs (Figures 3A,B and Supplementary Figure 3A). Notably, the oxidative stress induced by Vc treatment was largely prevented when ROS inhibitors, including 5 mM *N*-acetyl-L-cysteine (NAC) or GSH, were added (Figures 3A,B). We also measured the reduced GSH/oxidized GSH ratios (GSH/GSSG ratios) and levels of ATP production in both iRBCs and parasites derived from iRBCs. Consistently, Vc treatment decreased the GSH/GSSG ratio (Figure 3C) and ATP production in iRBCs (Figure 3D). In isolated parasites, oxidative pressure was consistent, judging by much lower levels of GSH, and Vc treatment caused a minor but reproducible decrease of the GSH/GSSG ratio (Figure 3C). ATP levels in isolated parasites, even though much lower than that in iRBCs, had a more prominent decrease when treated with Vc (Figure 3D). Taken together, these results confirm that high-dose Vc triggers oxidative stress in iRBCs, even more so in RBC-hosted parasites.

### Vc Promotes Eryptosis in iRBCs and Apoptosis in Parasites

**FIGURE 4:**
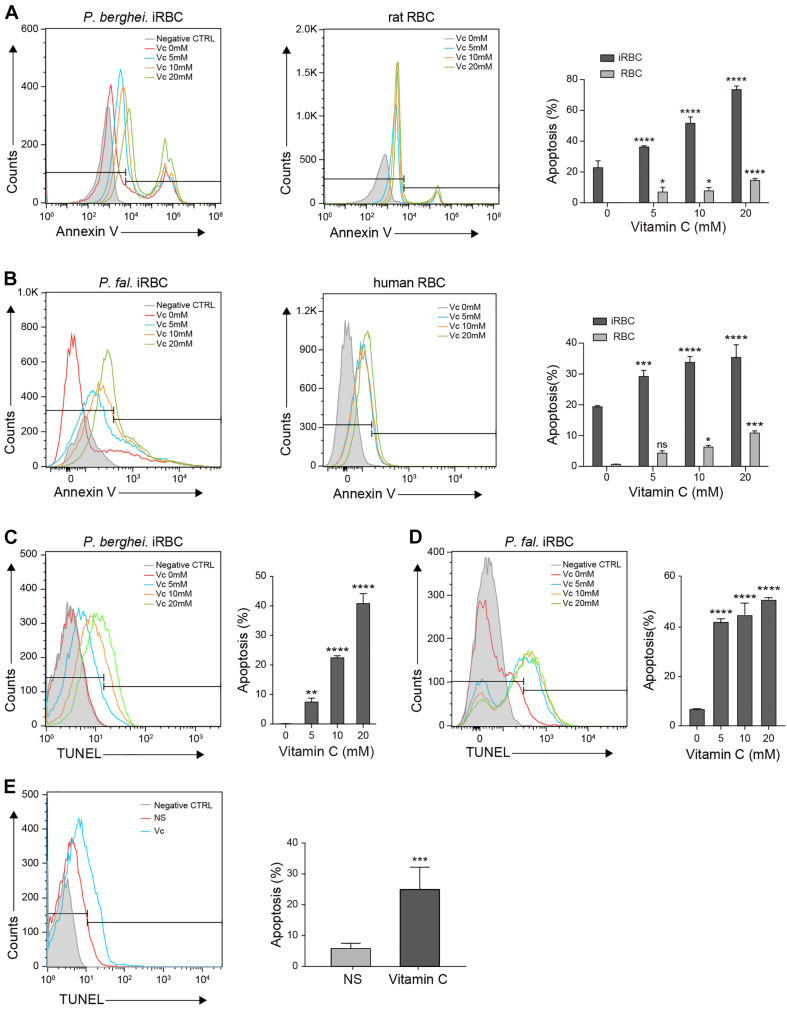
Vitamin C promotes apoptosis in *Plasmodium*-infected RBCs and parasites. **(A)** Vc induced significant apoptosis in *P. berghei–*infected RBCs and mild apoptosis in uninfected normal RBCs. Left: Representative FACS plots of iRBCs labeled with annexin V–FITC. Middle: Representative FACS plots of RBCs labeled with annexin V–FITC. Right: Quantitative analysis of apoptosis in iRBCs and RBCs treated with different doses of Vc (*n* = 3/group). Differences between Vc-treated iRBCs/RBCs and non-treated iRBCs/RBCs were analyzed by two-way ANOVA with Tukey multiple comparisons. **(B)** Vc induced significant apoptosis in *P. falciparum–*infected RBCs and mild apoptosis in uninfected normal RBCs. Left: Representative FACS plots of iRBCs labeled with annexin V–FITC. Middle: Representative FACS plots of RBCs labeled with annexin V–FITC. Right: Quantitative analysis of apoptosis in iRBCs and RBCs treated with different doses of Vc (*n* = 3/group). Differences between Vc-treated iRBCs/RBCs and non-treated iRBCs/RBCs were analyzed as described in **(A)**. **(C)** Vc induced apoptosis in *P. berghei* parasites. Representative FACS plots of iRBCs stained with TUNEL are displayed in the left panel. Quantitative analysis of apoptosis in *P. berghei* parasites is shown in the right panel, and the non-treated group was used as a control (*n* = 3/group). Data were analyzed by one-way ANOVA. **(D)** Vc induced apoptosis in *P. falciparum* parasites. Representative FACS plots of iRBCs stained with TUNEL are displayed in the left panel. Quantitative analysis of apoptosis in *P. falciparum* parasites is shown in the right panel, and the non-treated group was used as a control (*n* = 3/group). One-way ANOVA was performed. **(E)** Vc induced apoptosis in parasites cultured *in vivo*. *P. berghei*–infected mice were treated with NS or 4 g/kg Vc from day 0 for 3–4 days until parasitemia reached 2%. Isolated parasites were analyzed by TUNEL assay (*n* = 6 mice/group). The results were analyzed by unpaired two-tailed *t* test. Data are expressed as mean ± SD and representative of three independent experiments. *P < 0.05, **P < 0.01, ***P < 0.001, ****P < 0.0001, and n.s., not significant.

Extensive oxidative stress triggers apoptosis (Circu and Aw, 2010). Because of the absence of organelles, mature erythrocyte undergoes a form of programmed cell death termed *eryptosis*, which shares many similarities with apoptosis and is characterized by cell shrinkage, exposure of phosphatidylserine at the cell surface, and membrane blebbing (Lang et al., 2005). However, RBCs are more tolerant to such stress than other cells (Agar et al., 1986; Winterbourn and Stern, 1987). Therefore, we tested whether high-dose Vc increases eryptosis in iRBCs. First, we used annexin V, which detects cell surface phosphatidylserine, to compare eryptosis levels between RBCs and iRBCs after treatment with increasing concentrations of Vc. The iRBCs exhibited more apoptosis than RBCs, even in the absence of Vc (Figures 4A,B). High-dose Vc induced some apoptosis in uninfected cells, but caused significantly more damage to *P. berghei–*infected cells (Figure 4A) or *P. falciparum–*infected cells (Figure 4B). Next, we treated iRBCs with Vc, isolated the parasites, and measured apoptotic DNA breaks by TUNEL assay. High-dose Vc caused significant *P. berghei* parasite apoptosis (Figure 4C and Supplementary Figure 3B). The same results were obtained when *P. falciparum* parasites were tested (Figure 4D) and when mitochondrial membrane potentials were assessed (Supplementary Figure 3C). Finally, *P. berghei–*infected mice were injected with saline or Vc for 3–4 days. The parasites isolated from iRBCs treated with Vc exhibited more apoptosis than iRBCs treated with saline (Figure 4E). These results indicate that high-dose Vc selectively triggers apoptosis in both iRBCs and parasites.

### Vc Alleviates Physical Signs and Biochemical Indicators of Malaria

**FIGURE 5:**
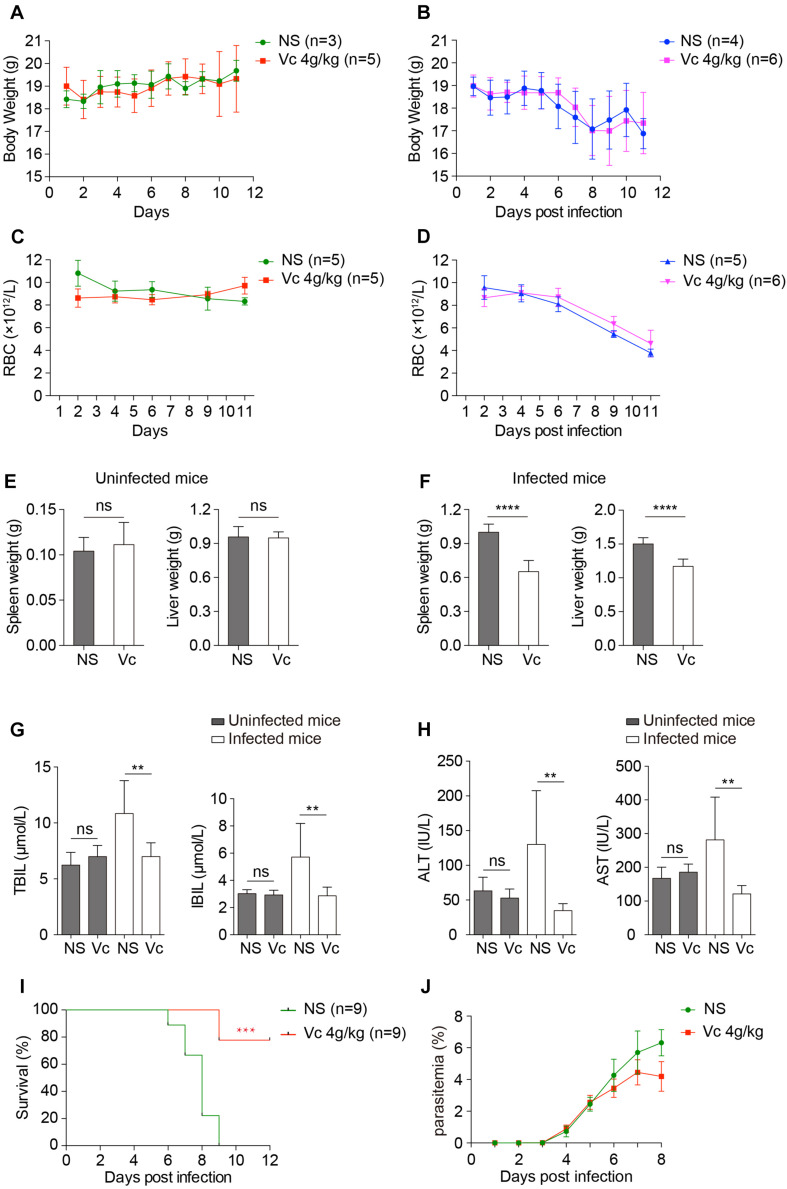
Vitamin C treatment improves the health conditions of infected mice and inhibits cerebral malaria development. **(A, B)** Vc treatment did not influence body weight in uninfected mice **(A)** or *P. berghei*–infected mice **(B)**. **(A):** NS vs. Vc, n.s. **(B):** NS vs. Vc, n.s. **(C, D)** Vc treatment did not influence total RBC number in uninfected mice **(C)** or *P. berghei*–infected mice **(D)**. **(C):** NS vs. Vc, n.s. **(D):** NS vs. Vc, n.s. Two-way ANOVA was performed in **(A–D)**. **(E)** Vc treatment at 4 g/kg for 12 days did not influence spleen and liver weight in normal uninfected mice (*n* = 5 mice/group). **(F)** Vc treatment 4 g/kg remarkably alleviated hepatosplenomegaly on day 11 p.i. compared to the NS-treated group (*n* = 5–7 mice/group). **(G)** Daily treatment of 4 g/kg Vc significantly decreased TBIL and IBIL serum levels in infected mice but had no influence on uninfected mice (*n* = 10/group). **(H)** Daily treatment of 4 g/kg Vc significantly decreased ALT and AST serum levels in infected mice but had no influence on uninfected mice (*n* = 10/group). Results in **(E–H)** were analyzed by unpaired two-tailed *t* test. **(I)** Vc treatment significantly inhibited cerebral malaria development in C57BL/6 mice. Survival rates between two groups were compared by the log-rank test. **(J)** Parasitemia of mice in **(I)**. Data are presented as mean ± SD and representative of three repetitions. **P < 0.01, ***P < 0.001, ****P < 0.0001, and n.s., not significant.

We analyzed the overall health conditions of *P. berghei–*infected mice treated with Vc. As determined above, 4 g/kg of Vc was injected daily. In neither normal nor infected mice, continuous administration of Vc did not influence body weight (Figures 5A,B) or the total number of RBCs (Figures 5C,D), indicating that Vc treatment did not impair the health status of mice. It was reported previously that Vc treatment regulates hematopoietic stem cell function (Agathocleous et al., 2017). Consistently, minor increases in RBC counts were seen after Vc treatment was applied to either healthy or *P. berghei–*infected mice (Figures 5C,D). Nevertheless, *P. berghei* infection caused anemia that was too severe to be overcome by Vc-induced RBC production (Figure 5D) and damaged the liver and spleen. We found that Vc treatments do not induce changes to the spleen and liver weight of uninfected mice (Figure 5E and Supplementary Figure 4), but reduce swelling in both organs (Figure 5F and Supplementary Figure 4). The total bilirubin (TBIL) and indirect bilirubin (IBIL), which are indicators of hemolysis and liver function, were increased in infected mice but restored to normal levels with Vc injections (Figure 5G). Similarly, liver malfunction, as indicated by alanine aminotransferase (ALT) and aspartate transaminase (AST) activities, was increased in infected mice and alleviated upon Vc treatment (Figure 5H). Notably, daily injections of high-dose Vc did not alter TBIL, IBIL, ALT, or AST in uninfected mice (Figures 5G, H), suggesting that the dose is tolerable and relatively safe.

We also investigated the effect of Vc on experimental cerebral malaria (ECM), a life-threatening form of malaria. Vc treatment reduced the incidence of ECM in *P. berghei–*infected C57BL/6 mice with relatively low parasitemia (Figures 5I,J). Taken together, these results suggest that high-dose Vc improves the health of *Plasmodium*-infected mice with no obvious signs of toxicity.

### Vc Acts Differently Than Chloroquine and Artemisinin

**FIGURE 6:**
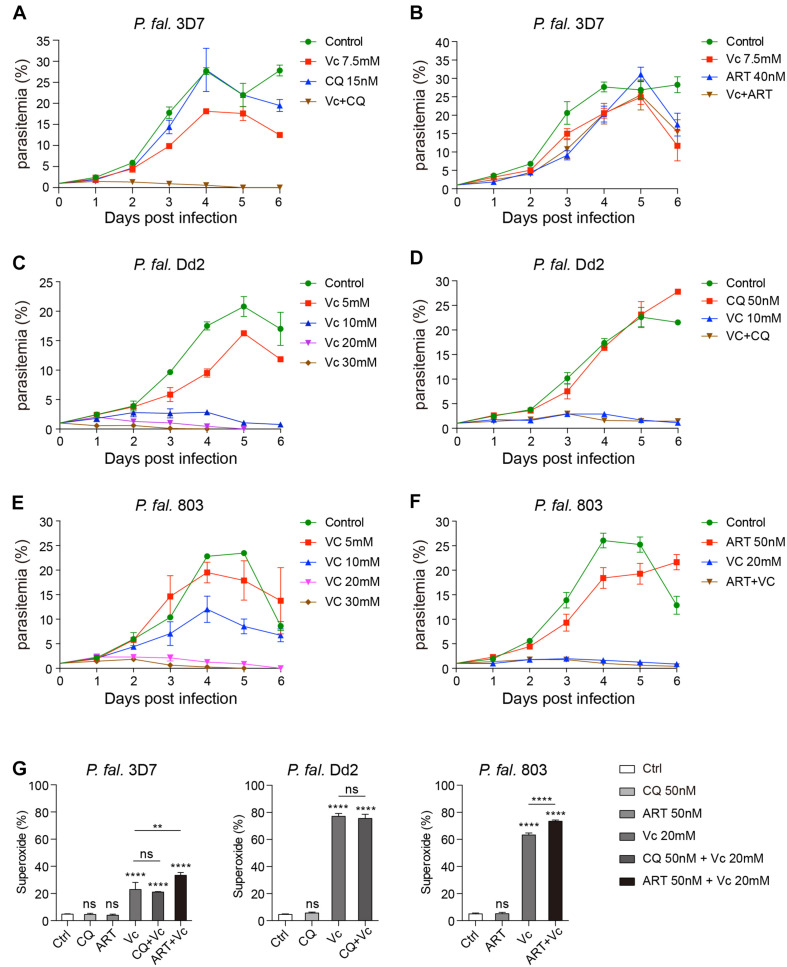
Vitamin C inhibits chloroquine- and artemisinin-resistant *P. falciparum* growth through induction of oxidative stress. **(A)** Vc had a synergistic effect with chloroquine (CQ) on *P. falciparum* 3D7 growth inhibition *in vitro*. Compared to the control group and the 7.5 mM Vc or 15 nM CQ treatment groups, the parasite growth was remarkably inhibited when treated with both 7.5 mM Vc and 15 nM CQ (*P* < 0.0001). **(B)** Combined treatment with Vc and artemisinin (ART) limited parasite growth inhibition. Vc + ART vs. Control: *P* < 0.01, Vc + ART vs. Vc 7.5 mM/ART 40 nM: n.s. **(C)** Vc significantly inhibited the growth of CQ-resistant *P. falciparum* Dd2. Control vs. Vc 5 mM/10 mM/20 mM/30 mM: *P* < 0.0001. **(D)** CQ did not influence the treatment effect of Vc in *P. falciparum* Dd2. Control vs. CQ 50 nM: n.s., control vs. Vc 10 mM/Vc + CQ: *P* < 0.0001, Vc 10 mM vs. Vc + CQ: n.s. **(E)** Vc significantly inhibited the growth of ART-resistant *P. falciparum* 803. Control vs. Vc 5 mM: n.s., control vs. Vc 10 mM/20 mM/30 mM: *P* < 0.0001. **(F)** ART did not influence the treatment effect of Vc in *P. falciparum* 803. Control vs. ART 50 nM: *P* < 0.01, control vs. Vc 20 mM/Vc + ART: *P* < 0.0001, Vc 20 mM vs. Vc + ART: n.s. The three *P. falciparum* strains were cultured with a starting parasitemia of 1% on day 0. All drugs were administered from day 0. The indicated dose of Vc and ART was administered for 3 h, whereas CQ was administered for 24 h from day 0. Parasitemia was determined daily before drug treatment. Differences between each drug treatment group and the control group were analyzed by two-way ANOVA with Tukey multiple comparisons. **(G)** Vc induced mitochondrial superoxide production in *P. falciparum* 3D7, chloroquine-resistant *P. falciparum* Dd2 and artemisinin-resistant *P. falciparum* 803 parasites cultured *in vitro* (*n* = 3/group). The superoxide production of *P. falciparum*–infected RBCs was measured by MitoSOX Red, and analyzed by one-way ANOVA. Data shown as mean ± SD. **P < 0.01, ****P < 0.0001, and n.s., not significant.

As Vc alone cannot eliminate *P. berghei* completely, we investigated the effect of Vc combined with other classical antimalaria drugs. First, we examined the minimal effective dose of chloroquine (CQ) and artemisinin (ART) in *in vitro* cultured *P. falciparum* 3D7. We found that 15 nM CQ or 20 nM ART were the minimal effective doses, and these doses had a nearly complete inhibitory effect on parasite growth (Supplementary Figures 5A,B). A synergistic effect was observed combining Vc and CQ, but not ART. Notably, high-dose Vc did not compromise the effects of CQ or ART (Figures 6A,B). The results suggest that Vc could potentially be applied with other antimalarial drugs.

To further investigate the effects of Vc on drug-resistant *P. falciparum*, we chose the Dd2 strain (CQ-resistant) and 803 strain (ART-resistant). The minimal effective dose of CQ in Dd2 is 150 nM (Supplementary Figure 5C), and that of ART in 803 is 50 nM (Supplementary Figure 5D), both of which are much higher than that used with 3D7. Consistently, Vc could inhibit Dd2 strain growth (Figure 6C), and CQ did not influence the effect of Vc when administered together (Figure 6D). Similarly, Vc inhibited 803 growth (Figure 6E), with no apparent interference by ART (Figure 6F). These results indicate that Vc-mediated inhibition of parasite growth is independent of the CQ or ART mechanisms.

To test whether CQ or ART induces equivalent oxidative stress as Vc, we monitored the superoxide production by drug-treated iRBCs using MitoSOX Red. Vc treatment significantly increased superoxide levels in all three *P. falciparum* strains (Figure 6G). In contrast, no detectable increase in oxidative species was observed when CQ or ART was used (Figure 6G), and they did not inhibit Vc-triggered oxidative stress (Figure 6G). These results confirm that Vc acts through mechanisms that are different to CQ and ART.

## Discussion

Our results indicate a previously unidentified activity of Vc to selectively inhibit blood-stage *Plasmodium* parasites *in vitro* and *in vivo*. The preference of Vc for iRBCs is attributed to their high demand for glucose. RBCs lack mitochondria, and the parasites do not express a mitochondrial pyruvate dehydrogenase (Foth et al., 2005); thus, both RBCs and the parasites rely completely on glucose for energetic metabolism. Mimicking glucose, DHA pours into iRBCs through the GLUT transporters on the cell surface. Our new findings suggest that *Plasmodium* HT likely acts similar to GLUTs, capable of taking up glucose and DHA. *P. falciparum* HT effectively transports glucose and fructose (Woodrow et al., 2000), the latter more structurally resembling Vc, suggesting a high probability of *Plasmodium* HT recognizing oxidized Vc.

Intracellular metabolism of the absorbed solute by iRBC can be complicated (Kirk et al., 2009). In this case, the overwhelming uptake of Vc mostly demands equally high reducing power in the cytosol of RBCs, and likely the cytosol of the parasites, because absorbed DHA is known to be quickly reduced to ascorbic acid (Vera et al., 1994; Yun et al., 2015). As expected, this causes oxidative stress in both the host cells and invading parasites. RBCs are capable of handling certain levels of oxidative pressure (Agar et al., 1986; Winterbourn and Stern, 1987). When treated with high-dose Vc, RBCs exhibit a low level of eryptosis, likely via elevated cytosolic oxidation. Importantly, iRBCs are much more sensitive to the stress posed by high-dose Vc than uninfected RBCs, as the parasites also cause oxidative damage during malarial pathogenesis (Percario et al., 2012). Conversely, the parasites are sensitive to oxidative stress caused by antimalarial oxidant drug exifone (Mahajan et al., 2005) or altered nutrient uptake by the host (Mancio-Silva et al., 2017; Zuzarte-Luis et al., 2017). The drastically increased oxidation of parasites subjected to high-dose Vc is likely caused indirectly by the oxidative environment of the RBC cytosol, directly by DHA uptake, or a combination of these effects. It is not clear whether the oxidation observed with Vc treatment directly triggers eryptosis. However, it can be eliminated by antioxidants, such as NAC, *in vitro*. Similar effects have been seen *in vivo*, which eventually blocks apoptosis induced by oxidative stress (Wang et al., 2018; Meng et al., 2020). Interestingly, high levels of oxidative stress are not seen with commonly used antimalaria drugs, including CQ and ART. These findings indicate a specific working mechanism for Vc and a potential for combined application.

Vitamin C has been used to alleviate oxidative damage caused by *Plasmodium* infection (Isah and Ibrahim, 2014) or to potentiate oxidant drugs (Isah and Ibrahim, 2014). However, the effects of Vc alone are minor, and the dosage usually much lower than used here. Relatively high Vc dose was recently shown to slowly inhibit *P. falciparum* growth *in vitro* (Wezena et al., 2017), but no mechanism was identified. We proposed that high-dose Vc inhibits *Plasmodium* growth by inducing oxidative stress. Given the natural resistance to oxidative pressure in RBCs, high dosage of Vc is necessary to tip the redox balance. The sufficient uptake of DHA by iRBCs is realized by the much-elevated demand for glucose upon parasite infection. Vc is considered as reducing agent, but the necessary conversion to an oxidized form during transmembrane transport prevents a direct impact on the redox system in uninfected cells. As expected, treatment with another reducing agent, DTT, caused hemolysis (Wezena et al., 2017). In our experiments, high-dose Vc did not cause significant hemolysis and liver damage; instead, it reduced swelling in the spleen caused by parasite infection and, more importantly, the occurrence of cerebral malaria. We also found that a similar antimalaria effect can be achieved by qod injection of Vc. Importantly, a plasma concentration of 20 mM Vc has been attempted in clinical trials for cancer therapy. In these cases, patients were subjected to intravenous (IV) ascorbate infusions three times a week for approximately 7 weeks, and this dosage is safe and well-tolerated (Schoenfeld et al., 2017). Furthermore, it is proposed that Vc can cause oxidative stress by disrupting iron metabolism in cancer cells (Schoenfeld et al., 2017). Similar mechanism is possible in Vc-treated iRBCs (Zhang et al., 2018), but needs further investigation.

Our results implicate a need for clinical trials of using high-dose Vc in malaria treatment. A daily IV injection of Vc would be expected to alleviate malaria infection in early stages. More importantly, prevention of cerebral malaria, if effective by Vc treatment, would be very beneficial. Given that Vc treatment targets the redox system in iRBC directly, risks for drug resistance would be low. In addition, Vc treatment can be promising even when *Plasmodium* develops resistance to commonly used drugs, including CQ and ART. While a recurrent IV administration poses risks for other infections and complicates the therapy, the low price for Vc with existing manufacture capability is appealing. Another concern of Vc-mediated *Plasmodium* inhibition is individuals with glucose-6-phosphate dehydrogenase (G-6-PD) deficiency, an observed genetic trait in malaria-endemic regions (Cappellini and Fiorelli, 2008). Administration of high-dose Vc in these individuals may lead to hemolytic anemia. Even though G-6-PD–deficient patients are easily diagnosed in wealthy regions and less susceptible to malarial infection (Cappadoro et al., 1998), such issue needs to be noted in regions where resources are very limited. Taken together, our findings offer a novel way of inducing selective cell death during malarial infection.

## Materials and Methods

### Ethics Statement

This study was approved by the Tianjin Medical University (TMU) Ethics Review Committee and the Tianjin Central Hospital of Gynecology Obstetrics Ethics Review Committee. All animal work was conducted according to Laboratory Animal Guidelines for Ethical Review of Animal Welfare (the National Standard of the People’s Republic of China, GB/T 35892-2018) and was approved by the Institutional Animal Care and Use Committee of TMU.

### Experimental Animals and Parasites

Male Wistar rats (80–100 g) and 6- to 8-week-old male BALB/c mice and female C57BL/6 mice were purchased from the Laboratory Animal Center of the Academy of Military Medical Sciences. All animals were bred at the TMU animal facility. Blood-stage *P. berghei* ANKA strain and *P. falciparum* 3D7 strain, Dd2 strain (CQ-resistant), and 803 strain (ART-resistant) were stored as stabilates in liquid nitrogen (Dd2 and 803 kindly provided by Dr. Lubin Jiang at Institut Pasteur of Shanghai, CAS). Parasitemia was examined by Giemsa-stained thin blood smears.

### *Plasmodium* Culture and Vc Administration

BALB/c mice were infected intravenously with 1.5 × 10^6^ *P. berghei–*iRBCs. These mice were euthanized 11–12 dpi or after 20 dpi. Vc (Sigma) was prepared as a 1 M working solution in 0.9% NaCl [normal saline (NS)] adjusted to pH 7.0. Mice were injected intraperitoneally with Vc (100 mg/kg, 2 g/kg, or 4 g/kg) or NS once a day.

*Plasmodium falciparum* was cultured in human erythrocytes (obtained from Tianjin Central Hospital of Gynecology Obstetrics) in RPMI 1640 medium (containing D-glucose at 2 g/L, ∼11 mM) supplemented with 25 mM HEPES, 0.5% AlbuMAX II, 100 μM hypoxanthine, and 12.5 μg/mL gentamicin as described previously (Cranmer et al., 1997). The initial parasitemia in each group was adjusted to 1%. The 1 M Vc was diluted to a 250 mM working solution. CQ was prepared as a 10 mM stock solution in NS. ART was prepared as a 10 mM stock solution in dimethyl sulfoxide. The cultured parasites were treated daily with Vc and/or ART for 3 h and then replaced with complete medium or treated with CQ for 24 h. When Vc and ART were combined, the parasites were treated with these drugs for 3 h, and then the complete medium was changed to only contain ART.

### Vc Uptake Assay

*Plasmodium berghei–*parasitized rat erythrocytes were purified using density gradient medium Nycodenz. *P. falciparum–*iRBCs were isolated using Percoll density gradient media. The parasites were isolated from iRBCs using 0.2% saponin. Harvested cells were incubated in medium containing 20% fetal bovine serum.

For Vc uptake assays in *P. berghei*, [^14^C]-Vc were added to the medium at a final concentration of 0.5 mM (0.5 μCi; specific activity: 8.5 mCi/mmol; PerkinElmer, Waltham, MA, United States). RBCs and iRBCs were incubated with 0.5 mM [^14^C]-Vc for 0.5 h and then lysed with 0.2% sodium dodecyl sulfate to measure the scintillation counts per minute (CPM). For parasite uptake, parasites were isolated from iRBCs incubated with [^14^C]-Vc and lysed to measure CPM. iRBCs 4 × 10^7^ were pretreated with or without 120 μM WZB117 (Sigma) for 5 min and incubated with 0.5 mM [^14^C]-Vc for 0.5 h. The [^14^C]-Vc uptake was detected in intact iRBCs, cytosol, and isolated parasites from the same number of lysed iRBCs. For the Vc and DHA competition assay, 2 × 10^7^ iRBCs or parasites isolated from 4 × 10^7^ iRBCs were pretreated with 10 mM reduced GSH (Sigma) or 10 U/mL AO (Sigma) for 5 min before adding [^14^C]-Vc. Treated cells were collected immediately to measure CMP. Relative [^14^C]-Vc uptake in the control group [pretreated with 1 × phosphate-buffered saline (PBS)] was defined as 100%. To investigate the Vc transporters in RBCs and parasites, they were pretreated with 120 μM 2-fluoro-6-(m-hydroxybenzoyloxy) phenyl m-hydroxybenzoate (WZB117) or 1 mM 3-*O*-(Undec-10-en)-yl-D-glucose (compound 3361) for 5 min before adding [^14^C]-Vc and measuring Vc uptake.

### Intracellular Oxidative Stress Analysis

We measured the relative molar ratios of reduced to oxidized GSH (GSH/GSSG) in 2 × 10^7^ iRBCs or parasites collected from 4 × 10^7^ iRBCs treated with Vc. The superoxide levels of iRBCs and RBCs were determined by measuring the mean fluorescence intensity of MitoSOX Red and DHE by flow cytometry. 2 × 10^7^ iRBCs or RBCs were pretreated with Vc, H_2_O_2_, menadione, or a combination of Vc and NAC/GSH for 1 h and then stained with 5 μM MitoSOX Red (Invitrogen, Carlsbad, CA, United States) for 15 min or 10 μM DHE (Beyotime, Shanghai, Beijing, China) for 30 min. Stained cells were analyzed on a BD FACSCanto II Flow Cytometer (BD Biosciences, San Jose, CA, United States). Data were analyzed using FlowJo software (TreeStar, Ashland, OR, United States).

### ATP Production Assay

To measure intracellular ATP, 2 × 10^7^ iRBCs were pretreated with Vc for 2 h. Parasites were isolated from pretreated iRBCs. The Enhanced ATP Assay Kit (Beyotime, Shanghai, Beijing, China) was used to measure ATP production in iRBCs and parasites following the manufacturer’s instructions. The detection range for the kit is 0.1 nM to 10 μM. ATP levels in non-treated cells were defined as 100%.

### TUNEL Assay

TUNEL assay was performed using the *in situ* Cell Death Detection Kit, Fluorescein (Roche, cat. no. 11684795910). Vc-treated or untreated iRBCs were fixed with 4% formaldehyde/PBS for 25 min and permeabilized with 0.2% Triton X-100. The cells were labeled by TUNEL mix for 60 min at 37°C in the dark. The labeled cells were analyzed by flow cytometry with a 488-nm argon-ion laser. *P. berghei–*infected mice were treated with NS or Vc from beginning day 0 for 3–4 days until parasitemia reached 2%. Isolated parasites were analyzed by TUNEL assay.

### Annexin V Apoptosis Assay

Apoptosis-associated phosphatidylserine exposure on the external leaflet of the membrane of RBCs and iRBCs was detected using the annexin V–fluorescein isothiocyanate (FITC) Apoptosis Detection Kit (Thermo Fisher Scientific). Cells were treated with Vc for 1 h, washed with 1 × PBS, and incubated in 200 μL 1 × binding buffer containing 5 μL annexin V–FITC for 5 min. The cells were washed with 1 × binding buffer and resuspended in binding buffer for flow cytometry.

### JC-1 Mitochondrial Membrane Potential Assay

Mitochondrial membrane potential of iRBCs was detected by MitoProbe^TM^ JC-1 Assay Kit (Thermo Fisher Scientific, #M34152). *P. berghei*–iRBCs 4 × 10^7^ were pretreated with different doses of Vc for 1 h, washed twice with 1 × PBS, and incubated in JC-1 (2 μM) at 37°C for 15 min. The labeled samples were washed twice with 1 × PBS and resuspended in FACS buffer to perform flow cytometer.

### Assessment of Health Status and Serum Biochemical Indices

Infected or uninfected mice were treated with NS or Vc daily from day 0 to 10 dpi and followed daily for body weight. Tail vein blood samples were collected every 2–3 days to determine the total number of RBCs. Blood samples were collected by retro-orbital bleeding after 11 days of treatment. Serum was collected to detect biochemical indices using a Roche P800 Clinical Biochemistry Analyzer (F. Hoffmann-La Roche Ltd., Basel, Switzerland), including TBIL, IBIL, ALT, and AST. The liver and spleen weights were also measured after 11-day treatment.

### Experimental Cerebral Malaria

To achieve ECM, female C57BL/6 mice were inoculated intravenously with 1 × 10^4^ *P. berghei* ANKA-iRBCs. Mice were treated daily with Vc (4 g/kg) or NS from day 0. Mice were followed daily for survival and neurological signs of ECM. Mice that developed ECM were euthanized when found moribund. At the end point of this experiment (12 dpi), the remaining mice were euthanized.

### Statistical Analysis

Data are presented as mean ± SD. Unpaired *t* test was used to compare two groups. To compare multiple groups, one-way analysis of variance (ANOVA) with Tukey test or Kruskal–Wallis ANOVA test was used. Two-way ANOVA with Tukey multiple-comparisons test was performed to compare the effect of multiple levels of two factors. Cumulative survival rates between two groups were plotted using the Kaplan–Meier method and compared by the log-rank test. All statistical analyses were performed using GraphPad software. *P* < 0.05 was considered significant: ^∗^*P* < 0.05, ^∗∗^*P* < 0.01, ^∗∗∗^*P* < 0.001, ^****^P < 0.0001, and n.s., not significant.

## Data Availability Statement

The original contributions presented in the study areincluded in the article/Supplementary Material, further inquiries can be directed to the corresponding author/s.

## Ethics Statement

The studies involving human participants were reviewed and approved by Tianjin Medical University (TMU) Ethics Review Committee and the Tianjin Central Hospital of Gynecology Obstetrics Ethics Review Committee. The patients/participants provided their written informed consent to participate in this study. The animal study was reviewed and approved by Institutional Animal Care and Use Committee of TMU.

## Author Contributions

QW and XS contributed to the overall study design. XS, MW, ZX, and YL performed most experiments, with help from MZ and LL. XS, ZX, MW, YL, MZ, and QW analyzed the data. XS and QW wrote the manuscript. All authors contributed to the article and approved the submitted version.

## Conflict of Interest

The authors declare that the research was conducted in the absence of any commercial or financial relationships that could be construed as a potential conflict of interest.

## Supplementary Material

Supplementary Figure 1 The inhibitory effects of vitamin C on *Plasmodium* growth. **(A)** Vc treatment inhibited *P. berghei* growth when administered from days 1 to 3 p.i. (*n* = 4–9 mice/group). Vc 4 g/kg was injected once a day starting from the different time points (days 0, 1, 2, and 3 p.i.). NS vs. Vc treatment from days 0/1/2/3 p.i.: *P* < 0.0001. Parasitemia of days 1–8 p.i. is shown in **(B)**. **(C)** Vc 4 g/kg alone cannot eliminate parasites infection in mice. Infected mice treated with NS (*n* = 10) died from day 15 p.i., and mice treated with Vc (*n* = 8) died from day 19 p.i. **(D)** Vc 3–5 mM significantly inhibited *P. falciparum* 3D7 growth when treated for 24 h daily from day 0 (parasitemia ∼1%; *n* = 3/group). Control vs. Vc 0.5 mM: n.s., control vs. Vc 3 mM: *P* < 0.01, control vs. Vc 5 mM: *P* < 0.0001. All data shown as mean ± SD are representative of at least three repetitions. Two-way ANOVA with Tukey multiple comparisons was used to analyze statistical differences. n.s., not significant.

Supplementary Figure 2 Vitamin C was absorbed by *P. falciparum* 3D7–infected RBCs, and absorption was inhibited by GLUT inhibitor WZB117 and HT inhibitor compound 3361. Relative [^14^C]-Vc uptakes are presented, with [^14^C]-Vc uptake in non-treated human erythrocytes defined as 100%. Data shown as mean ± SD are representative of three independent experiments and analyzed by one-way ANOVA. ^∗^*P* < 0.05, ^∗∗^*P* < 0.01, and n.s., not significant.

Supplementary Figure 3 Vitamin C induced oxidative stress in iRBCs and apoptosis in isolated *P. berghei* parasites. **(A)** Vc treatment induced mild superoxide production in normal rat RBCs measured by MitoSOX Red (left panel) and DHE (right panel). Oxidants H_2_O_2_ and menadione were used as positive controls. Kruskal–Wallis ANOVA test was used for the data of the left panel and one-way ANOVA was performed to analyze the data of the right panel. **(B)** Representative images of TUNEL assay in Figure 4C. **(C)** Vc treatment led to iRBC apoptosis determined by mitochondrial membrane potentials. Left panel showed the representative FACS plots. Right panel showed the ratio of JC-1 (red)/JC-1 (green) parasites populations (*n* = 3/group). Data shown as mean ± SD are analyzed by one-way ANOVA. ^∗∗^*P* < 0.01, ^∗∗∗^*P* < 0.001, ^****^*P* < 0.0001, and n.s., not significant.

Supplementary Figure 4 Vitamin C alleviated hepatosplenomegaly in *P. berghei*–infected mice, whereas it had no effect on the spleen and liver weights of uninfected normal mice. The quantitative analyses of spleen and liver weight were showed in Figures 6E,F.

Supplementary Figure 5 Effective therapeutic doses of chloroquine (CQ) and artemisinin (ART) in drug-sensitive or drug-resistant *P. falciparum* strains cultured *in vitro*. **(A)** The effective therapeutic dose of CQ in *P. falciparum* 3D7 was more than 15 nM. Control vs. CQ 10 nM: n.s., control vs. CQ 15 nM: *P* < 0.05, control vs. CQ 20 nM: *P* < 0.0001, control vs. CQ 25 nM: *P* < 0.0001. **(B)** The effective therapeutic dose of ART in *P. falciparum* 3D7 was more than 20 nM. Control vs. ART 20 nM: *P* < 0.01, control vs. ART 30 nM: *P* < 0.0001, control vs. ART 40 nM: *P* < 0.0001, control vs. ART 50 nM: *P* < 0.0001. **(C)** The effective therapeutic dose of CQ in *P. falciparum* Dd2 was more than 150 nM. Control vs. CQ 50 nM: n.s., control vs. CQ 100 nM: n.s., control vs. CQ 150 nM: *P* < 0.0001, control vs. CQ 200 nM: *P* < 0.0001. **(D)** The effective therapeutic dose of ART in *P. falciparum* 803 was more than 50 nM. Control vs. ART 20 nM: n.s., control vs. ART 30 nM: n.s., control vs. ART 40 nM: n.s., control vs. ART 50 nM: *P* < 0.0001, control vs. ART 60 nM: *P* < 0.0001. Data are mean ± SD and representative of three repetitions. Two-way ANOVA with Tukey multiple comparisons was performed to analyze the data. n.s., not significant.

## References

[B1] Agar N. S. Sadrzadeh S. M. Hallaway P. E. Eaton J. W. (1986). Erythrocyte catalase. A somatic oxidant defense? *J. Clin. Invest.* 77 319–321. 10.1172/JCI112294 3944256 PMC423343

[B2] Agathocleous M. Meacham C. E. Burgess R. J. Piskounova E. Zhao Z. Crane G. M. (2017). Ascorbate regulates haematopoietic stem cell function and leukaemogenesis. *Nature* 549 476–481. 10.1038/nature23876 28825709 PMC5910063

[B3] Altenberg B. Greulich K. O. (2004). Genes of glycolysis are ubiquitously overexpressed in 24 cancer classes. *Genomics* 84 1014–1020. 10.1016/j.ygeno.2004.08.010 15533718

[B4] Cameron E. Pauling L. (1976). Supplemental ascorbate in the supportive treatment of cancer: prolongation of survival times in terminal human cancer. *Proc. Natl. Acad. Sci. U.S.A.* 73 3685–3689. 10.1073/pnas.73.10.3685 1068480 PMC431183

[B5] Cameron E. Pauling L. (1978). Supplemental ascorbate in the supportive treatment of cancer: reevaluation of prolongation of survival times in terminal human cancer. *Proc. Natl. Acad. Sci. U.S.A.* 75 4538–4542. 10.1073/pnas.75.9.4538 279931 PMC336151

[B6] Cappadoro M. Giribaldi G. O’Brien E. Turrini F. Mannu F. Ulliers D. (1998). Early phagocytosis of glucose-6-phosphate dehydrogenase (G6PD)-deficient erythrocytes parasitized by Plasmodium falciparum may explain malaria protection in G6PD deficiency. *Blood* 92 2527–2534. 10.1182/blood.v92.7.2527.2527_2527_2534 9746794

[B7] Cappellini M. D. Fiorelli G. (2008). Glucose-6-phosphate dehydrogenase deficiency. *Lancet* 371 64–74. 10.1016/S0140-6736(08)60073-2 18177777

[B8] Chen Q. Espey M. G. Sun A. Y. Lee J. H. Krishna M. C. Shacter E. (2007). Ascorbate in pharmacologic concentrations selectively generates ascorbate radical and hydrogen peroxide in extracellular fluid in vivo. *Proc. Natl. Acad. Sci. U.S.A.* 104 8749–8754. 10.1073/pnas.0702854104 17502596 PMC1885574

[B9] Chen Q. Espey M. G. Sun A. Y. Pooput C. Kirk K. L. Krishna M. C. (2008). Pharmacologic doses of ascorbate act as a prooxidant and decrease growth of aggressive tumor xenografts in mice. *Proc. Natl. Acad. Sci. U.S.A.* 105 11105–11109. 10.1073/pnas.0804226105 18678913 PMC2516281

[B10] Circu M. L. Aw T. Y. (2010). Reactive oxygen species, cellular redox systems, and apoptosis. *Free Radic. Biol. Med.* 48 749–762. 10.1016/j.freeradbiomed.2009.12.022 20045723 PMC2823977

[B11] Cranmer S. L. Magowan C. Liang J. Coppel R. L. Cooke B. M. (1997). An alternative to serum for cultivation of *Plasmodium falciparum* in vitro. *Trans. R. Soc. Trop. Med. Hyg.* 91 363–365. 10.1016/s0035-9203(97)90110-3 9231219

[B12] Desai S. A. Bezrukov S. M. Zimmerberg J. (2000). A voltage-dependent channel involved in nutrient uptake by red blood cells infected with the malaria parasite. *Nature* 406 1001–1005. 10.1038/35023000 10984055

[B13] Foth B. J. Stimmler L. M. Handman E. Crabb B. S. Hodder A. N. McFadden G. I. (2005). The malaria parasite Plasmodium falciparum has only one pyruvate dehydrogenase complex, which is located in the apicoplast. *Mol. Microbiol.* 55 39–53. 10.1111/j.1365-2958.2004.04407.x 15612915

[B14] Isah M. B. Ibrahim M. A. (2014). The role of antioxidants treatment on the pathogenesis of malarial infections: a review. *Parasitol. Res.* 113 801–809. 10.1007/s00436-014-3804-1 24525759

[B15] Joet T. Eckstein-Ludwig U. Morin C. Krishna S. (2003). Validation of the hexose transporter of Plasmodium falciparum as a novel drug target. *Proc. Natl. Acad. Sci. U.S.A.* 100 7476–7479. 10.1073/pnas.1330865100 12792024 PMC164611

[B16] Kirk K. Horner H. A. Kirk J. (1996). Glucose uptake in Plasmodium falciparum-infected erythrocytes is an equilibrative not an active process. *Mol. Biochem. Parasitol.* 82 195–205. 10.1016/0166-6851(96)02734-x 8946385

[B17] Kirk K. Howitt S. M. Broer S. Saliba K. J. Downie M. J. (2009). Purine uptake in Plasmodium: transport versus metabolism. *Trends Parasitol.* 25 246–249. 10.1016/j.pt.2009.03.006 19423394

[B18] Lang K. S. Lang P. A. Bauer C. Duranton C. Wieder T. Huber S. M. (2005). Mechanisms of suicidal erythrocyte death. *Cell Physiol. Biochem.* 15 195–202. 10.1159/000086406 15956782

[B19] Linster C. L. Van Schaftingen E. (2007). Vitamin C. Biosynthesis, recycling and degradation in mammals. *FEBS J.* 274 1–22. 10.1111/j.1742-4658.2006.05607.x 17222174

[B20] Mahajan S. S. Kamath V. R. Ghatpande S. S. (2005). Synergistic antimalarial activity of ketones with rufigallol and vitamin C. *Parasitology* 131 459–466. 10.1017/S0031182005008267 16174410

[B21] Mancio-Silva L. Slavic K. Grilo Ruivo MT Grosso A. R. Modrzynska K. K. Vera I. M. (2017). Nutrient sensing modulates malaria parasite virulence. *Nature* 547 213–216. 10.1038/nature23009 28678779 PMC5511512

[B22] May J. M. Qu Z. C. Qiao H. Koury M. J. (2007). Maturational loss of the vitamin C transporter in erythrocytes. *Biochem. Biophys. Res. Commun.* 360 295–298. 10.1016/j.bbrc.2007.06.072 17586466 PMC1964531

[B23] Meireles P. Sales-Dias J. Andrade C. M. Mello-Vieira J. Mancio-Silva L. Simas J. P. (2017). GLUT1-mediated glucose uptake plays a crucial role during Plasmodium hepatic infection. *Cell Microbiol.* 19:e12646. 10.1111/cmi.12646 27404888 PMC5297879

[B24] Meng J. Lv Z. Zhang Y. Wang Y. Qiao X. Sun C. (2020). Precision redox: the key for antioxidant pharmacology. *Antioxid. Redox Signal.* [Epub ahead of print]. 10.1089/ars.2020.8212 33270507 PMC8080931

[B25] Montel-Hagen A. Blanc L. Boyer-Clavel M. Jacquet C. Vidal M. Sitbon M. (2008a). The Glut1 and Glut4 glucose transporters are differentially expressed during perinatal and postnatal erythropoiesis. *Blood* 112 4729–4738. 10.1182/blood-2008-05-159269 18796630

[B26] Montel-Hagen A. Kinet S. Manel N. Mongellaz C. Prohaska R. Battini J. L. (2008b). Erythrocyte Glut1 triggers dehydroascorbic acid uptake in mammals unable to synthesize vitamin C. *Cell* 132 1039–1048. 10.1016/j.cell.2008.01.042 18358815

[B27] Nakajima Y. Shantha T. R. Bourne G. H. (1969). Histochemical detection of L-gulonolactone: phenazine methosulfate oxidoreductase activity in several mammals with special reference to synthesis of vimin C in primates. *Histochemie* 18 293–301. 10.1007/bf00279880 4982058

[B28] Nguitragool W. Bokhari A. A. Pillai A. D. Rayavara K. Sharma P. Turpin B. (2011). Malaria parasite clag3 genes determine channel-mediated nutrient uptake by infected red blood cells. *Cell* 145 665–677. 10.1016/j.cell.2011.05.002 21620134 PMC3105333

[B29] Percario S. Moreira D. R. Gomes B. A. Ferreira M. E. Gonçalves A. C. Laurindo P. S. (2012). Oxidative stress in malaria. *Int. J. Mol. Sci.* 13 16346–16372. 10.3390/ijms131216346 23208374 PMC3546694

[B30] Pillai A. D. Nguitragool W. Lyko B. Dolinta K. Butler M. M. Nguyen S. T. (2012). Solute restriction reveals an essential role for clag3-associated channels in malaria parasite nutrient acquisition. *Mol. Pharmacol.* 82 1104–1114. 10.1124/mol.112.081224 22949525 PMC3502622

[B31] Pollock J. I. Mullin R. J. (1987). Vitamin C biosynthesis in prosimians: evidence for the anthropoid affinity of *Tarsius*. *Am. J. Phys. Anthropol.* 73 65–70. 10.1002/ajpa.1330730106 3113259

[B32] Schoenfeld J. D. Sibenaller Z. A. Mapuskar K. A. Wagner B. A. Cramer-Morales K. L. Furqan M. (2017). O2(-) and H2O2-mediated disruption of fe metabolism causes the differential susceptibility of NSCLC and GBM cancer cells to pharmacological ascorbate. *Cancer Cell* 31 487.e8–500.e8. 10.1016/j.ccell.2017.02.018 28366679 PMC5497844

[B33] Tsukaguchi H. Tokui T. Mackenzie B. Berger U. V. Chen X. Z. Wang Y. (1999). A family of mammalian Na+-dependent L-ascorbic acid transporters. *Nature* 399 70–75. 10.1038/19986 10331392

[B34] van Wijk R. van Solinge W. W. (2005). The energy-less red blood cell is lost: erythrocyte enzyme abnormalities of glycolysis. *Blood* 106 4034–4042. 10.1182/blood-2005-04-1622 16051738

[B35] Vera J. C. Rivas C. I. Fischbarg J. Golde D. W. (1993). Mammalian facilitative hexose transporters mediate the transport of dehydroascorbic acid. *Nature* 364 79–82. 10.1038/364079a0 8316303

[B36] Vera J. C. Rivas C. I. Zhang R. H. Farber C. M. Golde D. W. (1994). Human HL-60 myeloid leukemia cells transport dehydroascorbic acid via the glucose transporters and accumulate reduced ascorbic acid. *Blood* 84 1628–1634. 10.1182/blood.v84.5.1628.bloodjournal8451628 8068952

[B37] Wang B. Fu J. Yu T. Xu A. Qin W. Yang Z. (2018). Contradictory effects of mitochondria- and non-mitochondria-targeted antioxidants on hepatocarcinogenesis by altering DNA repair in mice. *Hepatology* 67 623–635. 10.1002/hep.29518 28898446

[B38] Wezena C. A. Krafczyk J. Staudacher V. Deponte M. (2017). Growth inhibitory effects of standard pro- and antioxidants on the human malaria parasite *Plasmodium falciparum*. *Exp. Parasitol.* 180 64–70. 10.1016/j.exppara.2017.02.017 28242353

[B39] Winterbourn C. C. Stern A. (1987). Human red cells scavenge extracellular hydrogen peroxide and inhibit formation of hypochlorous acid and hydroxyl radical. *J. Clin. Invest.* 80 1486–1491. 10.1172/JCI113230 2824562 PMC442408

[B40] Wood I. S. Trayhurn P. (2003). Glucose transporters (GLUT and SGLT): expanded families of sugar transport proteins. *Br. J. Nutr.* 89 3–9. 10.1079/BJN2002763 12568659

[B41] Woodrow C. J. Burchmore R. J. Krishna S. (2000). Hexose permeation pathways in Plasmodium falciparum-infected erythrocytes. *Proc. Natl. Acad. Sci. U.S.A.* 97 9931–9936. 10.1073/pnas.170153097 10954735 PMC27630

[B42] Yun J. Mullarky E. Lu C. Bosch K. N. Kavalier A. Rivera K. (2015). Vitamin C selectively kills KRAS and BRAF mutant colorectal cancer cells by targeting GAPDH. *Science* 350 1391–1396. 10.1126/science.aaa5004 26541605 PMC4778961

[B43] Zhang D. L. Wu J. Shah B. N. Greutélaers K. C. Ghosh M. C. Ollivierre H. (2018). Erythrocytic ferroportin reduces intracellular iron accumulation, hemolysis, and malaria risk. *Science* 359 1520–1523. 10.1126/science.aal2022 29599243 PMC8349187

[B44] Zuzarte-Luis V. Mello-Vieira J. Marreiros I. M. Liehl P. Chora A. F. Carret C. K. (2017). Dietary alterations modulate susceptibility to *Plasmodium infection*. *Nat. Microbiol.* 2 1600–1607. 10.1038/s41564-017-0025-2 28947801

