# Supplementary figures and images for: Vitamin C Inhibits Blood-Stage Plasmodium Parasites via Oxidative Stress
Source: Front Cell Dev Biol. 2021 May 11;9:639944. doi: 10.3389/fcell.2021.639944 (PMC8144511; doi:10.3389/fcell.2021.639944)

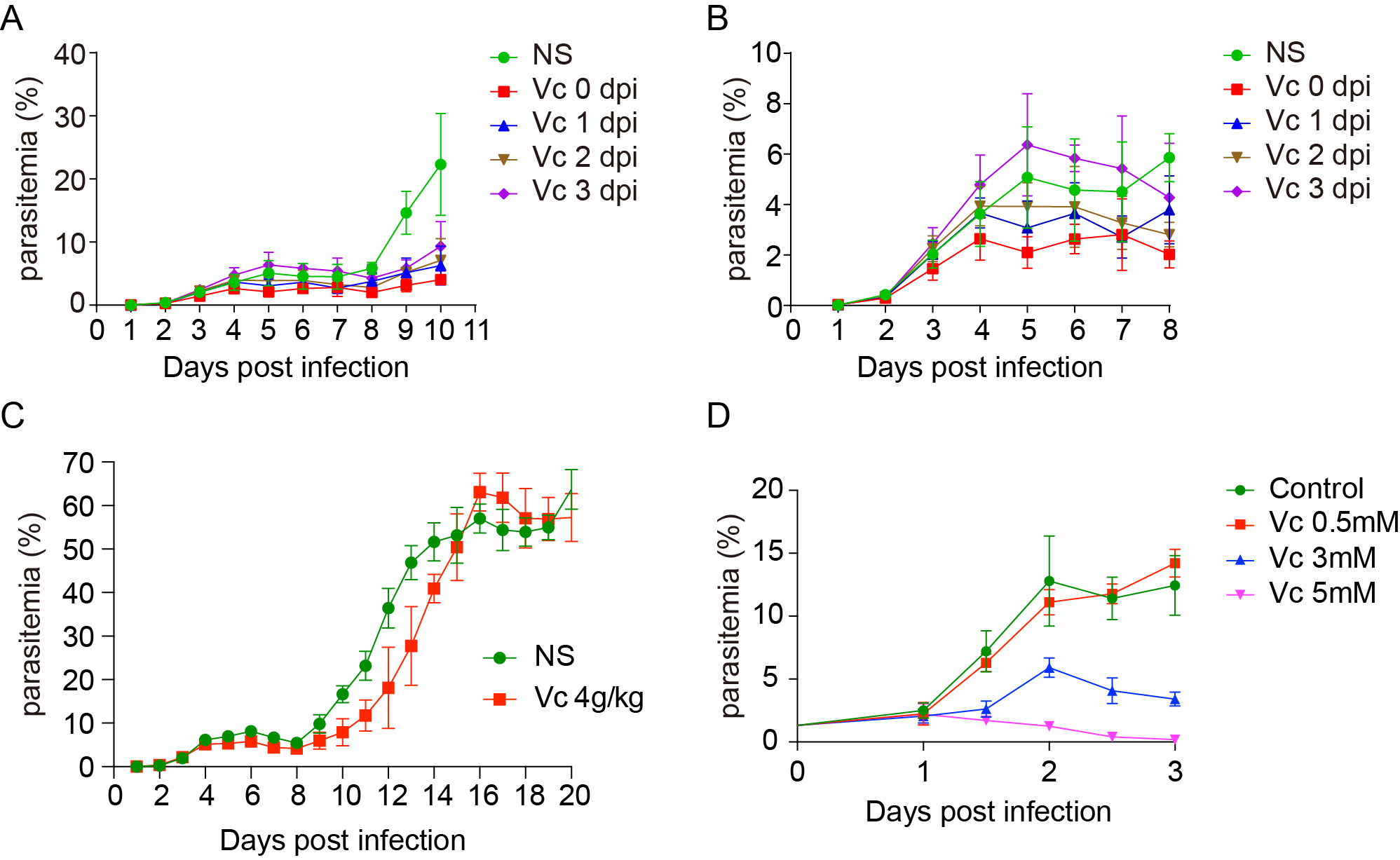

Supplement: Supplementary Figure 1 — The inhibitory effects of vitamin C on Plasmodium growth. (A) Vc treatment inhibited P. berghei growth when administered from days 1 to 3 p.i. (n = 4–9 mice/group). Vc 4 g/kg was injected once a day starting from the different time points (days 0, 1, 2, and 3 p.i.). NS vs. Vc treatment from days 0/1/2/3 p.i.: P < 0.0001. Parasitemia of days 1–8 p.i. is shown in (B). (C) Vc 4 g/kg alone cannot eliminate parasites infection in mice. Infected mice treated with NS (n = 10) died from day 15 p.i., and mice treated with Vc (n = 8) died from day 19 p.i. (D) Vc 3–5 mM significantly inhibited P. falciparum 3D7 growth when treated for 24 h daily from day 0 (parasitemia ∼1%; n = 3/group). Control vs. Vc 0.5 mM: n.s., control vs. Vc 3 mM: P < 0.01, control vs. Vc 5 mM: P < 0.0001. All data shown as mean ± SD are representative of at least three repetitions. Two-way ANOVA with Tukey multiple comparisons was used to analyze statistical differences. n.s., not significant. [file Image_1.jpg]

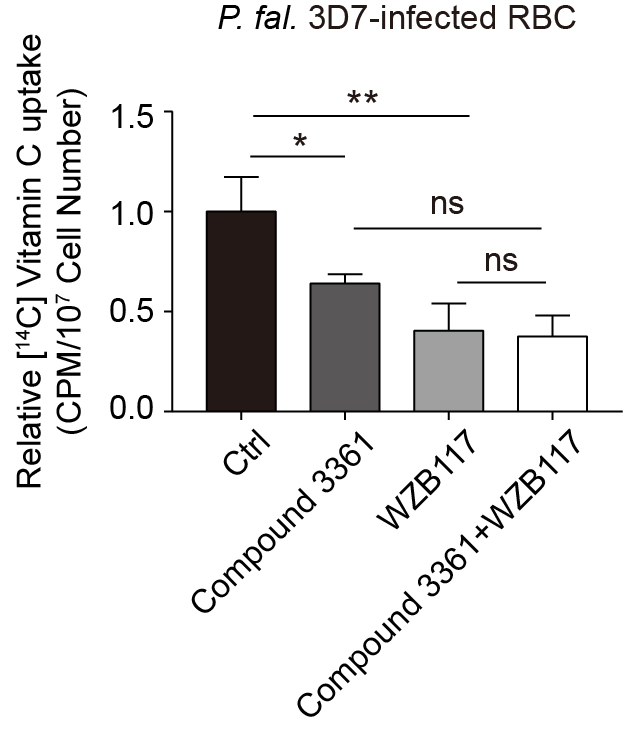

Supplement: Supplementary Figure 2 — Vitamin C was absorbed by P. falciparum 3D7–infected RBCs, and absorption was inhibited by GLUT inhibitor WZB117 and HT inhibitor compound 3361. Relative [14C]-Vc uptakes are presented, with [14C]-Vc uptake in non-treated human erythrocytes defined as 100%. Data shown as mean ± SD are representative of three independent experiments and analyzed by one-way ANOVA. ∗P < 0.05, ∗∗P < 0.01, and n.s., not significant. [file Image_2.jpg]

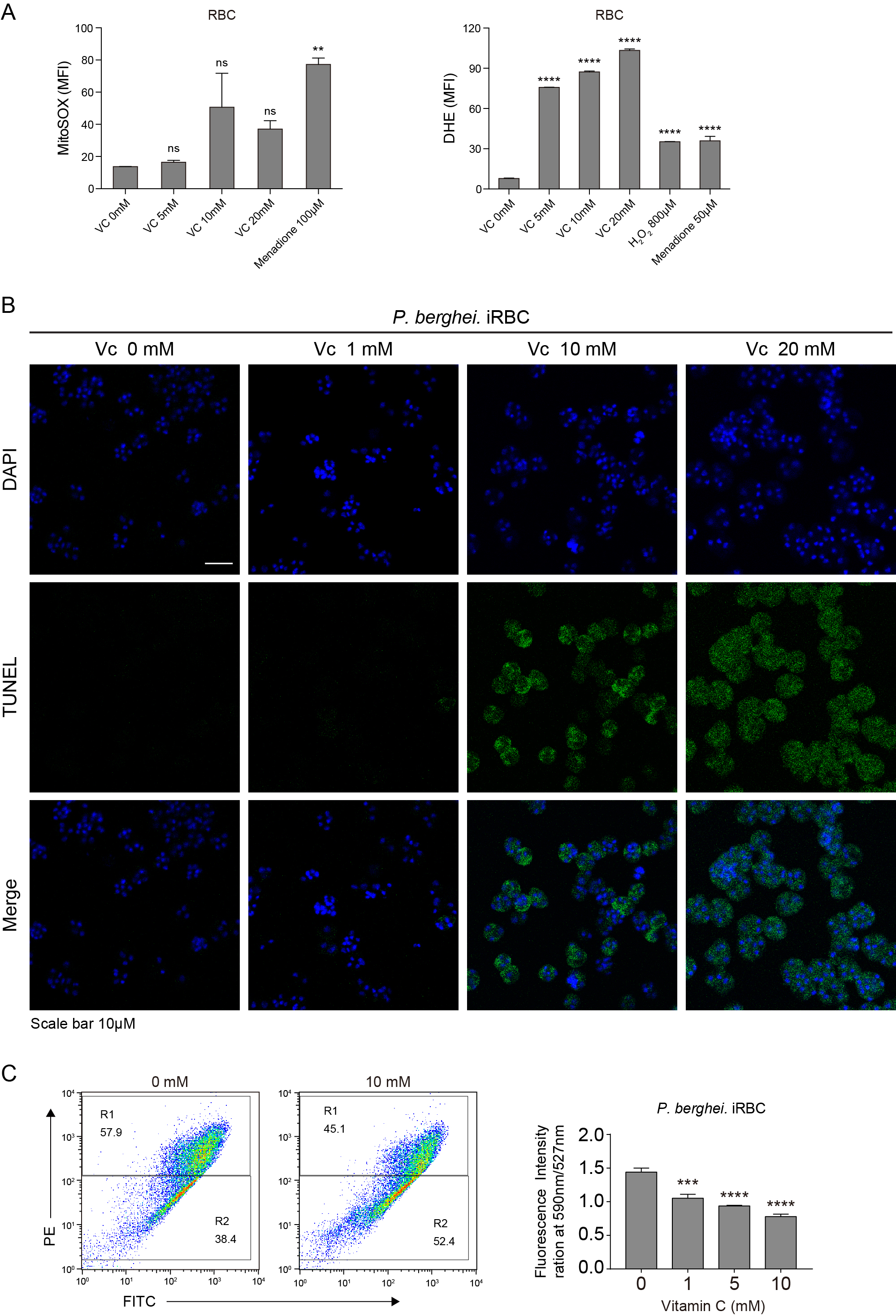

Supplement: Supplementary Figure 3 — Vitamin C induced oxidative stress in iRBCs and apoptosis in isolated P. berghei parasites. (A) Vc treatment induced mild superoxide production in normal rat RBCs measured by MitoSOX Red (left panel) and DHE (right panel). Oxidants H2O2 and menadione were used as positive controls. Kruskal–Wallis ANOVA test was used for the data of the left panel and one-way ANOVA was performed to analyze the data of the right panel. (B) Representative images of TUNEL assay in Figure 4C. (C) Vc treatment led to iRBC apoptosis determined by mitochondrial membrane potentials. Left panel showed the representative FACS plots. Right panel showed the ratio of JC-1 (red)/JC-1 (green) parasites populations (n = 3/group). Data shown as mean ± SD are analyzed by one-way ANOVA. ∗∗P < 0.01, ∗∗∗P < 0.001, ****P < 0.0001, and n.s., not significant. [file Image_3.jpg]

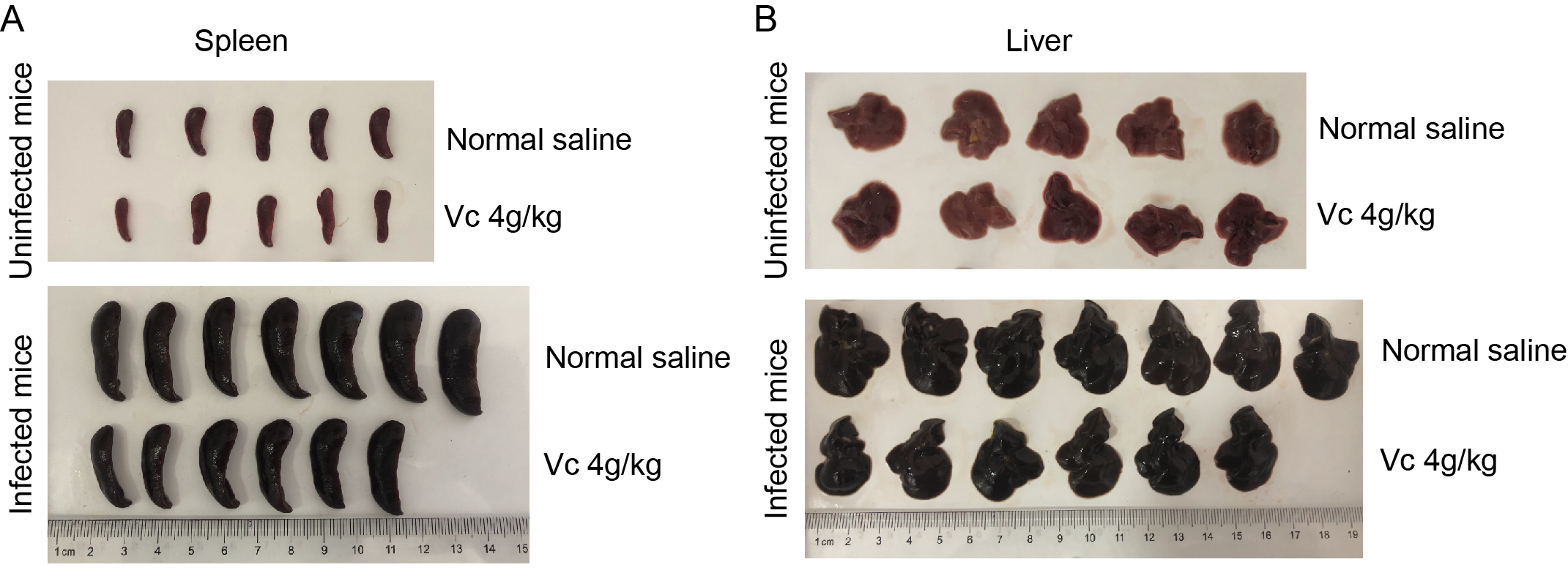

Supplement: Supplementary Figure 4 — Vitamin C alleviated hepatosplenomegaly in P. berghei–infected mice, whereas it had no effect on the spleen and liver weights of uninfected normal mice. The quantitative analyses of spleen and liver weight were showed in Figures 6E,F. [file Image_4.jpg]

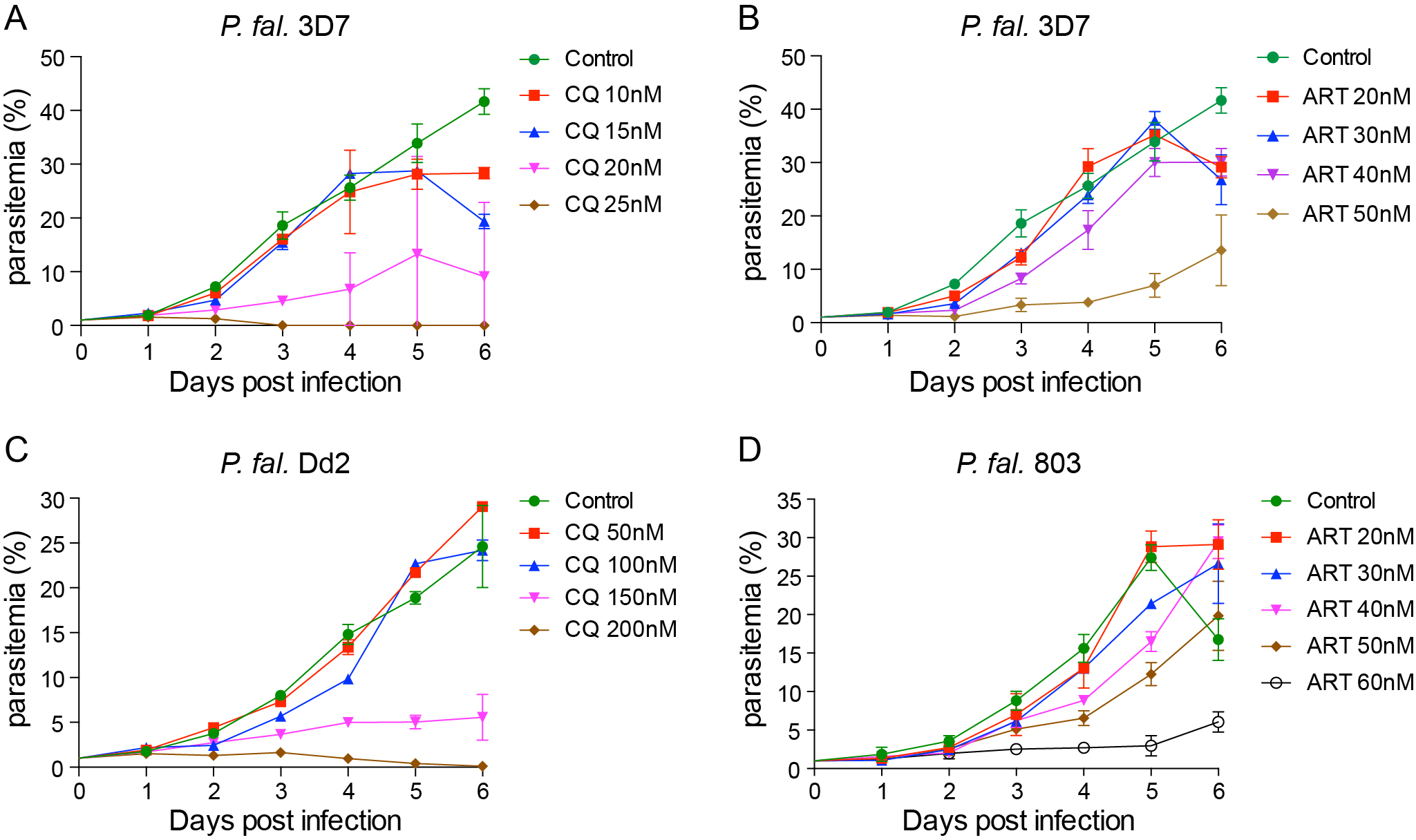

Supplement: Supplementary Figure 5 — Effective therapeutic doses of chloroquine (CQ) and artemisinin (ART) in drug-sensitive or drug-resistant P. falciparum strains cultured in vitro. (A) The effective therapeutic dose of CQ in P. falciparum 3D7 was more than 15 nM. Control vs. CQ 10 nM: n.s., control vs. CQ 15 nM: P < 0.05, control vs. CQ 20 nM: P < 0.0001, control vs. CQ 25 nM: P < 0.0001. (B) The effective therapeutic dose of ART in P. falciparum 3D7 was more than 20 nM. Control vs. ART 20 nM: P < 0.01, control vs. ART 30 nM: P < 0.0001, control vs. ART 40 nM: P < 0.0001, control vs. ART 50 nM: P < 0.0001. (C) The effective therapeutic dose of CQ in P. falciparum Dd2 was more than 150 nM. Control vs. CQ 50 nM: n.s., control vs. CQ 100 nM: n.s., control vs. CQ 150 nM: P < 0.0001, control vs. CQ 200 nM: P < 0.0001. (D) The effective therapeutic dose of ART in P. falciparum 803 was more than 50 nM. Control vs. ART 20 nM: n.s., control vs. ART 30 nM: n.s., control vs. ART 40 nM: n.s., control vs. ART 50 nM: P < 0.0001, control vs. ART 60 nM: P < 0.0001. Data are mean ± SD and representative of three repetitions. Two-way ANOVA with Tukey multiple comparisons was performed to analyze the data. n.s., not significant. [file Image_5.jpg]
